# Primary care consultation length by deprivation and multimorbidity in England: an observational study using electronic patient records

**DOI:** 10.3399/bjgp20X714029

**Published:** 2020-12-15

**Authors:** Anya Gopfert, Sarah R Deeny, Rebecca Fisher, Mai Stafford

**Affiliations:** Oxford University Hospitals, Oxford.; Data Analytics;; The Health Foundation, London.; The Health Foundation, London.

**Keywords:** England, mental health, multimorbidity, primary health care, referral and consultation, socioeconomic factors

## Abstract

**Background:**

Longer GP consultations are recommended as one way of improving care for people with multimorbidity. In Scotland, patients who are multimorbid and living in deprived areas do not have longer consultations, although their counterparts in the least deprived areas do. This example of the inverse care law has not been examined in England.

**Aim:**

To assess GP consultation length by socioeconomic deprivation and multimorbidity.

**Design and setting:**

Random sample of 1.2 million consultations from 1 April 2014 to 31 March 2016 for 190 036 adults in England drawn from the Clinical Practice Research Datalink.

**Method:**

Consultation duration was derived from time of opening and closing the patient’s electronic record. Mean duration was estimated by multimorbidity level and type, adjusted for number of consultations and other patient and staff characteristics and patient and practice random effects.

**Results:**

Consultations lasted 10.9 minutes on average and mean duration increased with number of conditions. Patients with ≥6 conditions had 0.9 (95% confidence interval [CI] = 0.8 to 1.0) minutes longer than those with none. Patients who are multimorbid and with a mental health condition had 0.5 (CI = 0.4 to 0.5) minutes longer than patients who were not multimorbid. However, consultations were 0.5 (CI = 0.4 to 0.5) minutes shorter in the most compared with the least deprived fifth of areas at all levels of multimorbidity.

**Conclusion:**

GPs in England spend longer with patients who have more conditions, but, at all multimorbidity levels, those in deprived areas have less time per GP consultation. Further research is needed to assess the impact of consultation length on patient and system outcomes for those with multimorbidity.

## INTRODUCTION

Multimorbidity is defined as the coexistence of ≥2 conditions within an individual. Prevalence estimates depend on the conditions counted but recent studies suggest that around 23–27% in the general population^1^^,^^2^ — an estimated 14.2 million people in England^3^ — are affected, and that prevalence is increasing across the UK.^4^ The risk of multimorbidity increases with advancing age and is strongly linked to socioeconomic position, occurring more frequently and 10–15 years earlier in the most deprived compared with the least deprived areas.^2^ Living with multimorbidity can be challenging, and may result in poor quality of life and difficulties with everyday activities.^5^^,^^6^ People with multimorbidity often require significant time and interaction with health services. Providing care to these individuals can be challenging because of the complexity of intersecting health and care requirements.^7^ In addition, around 30% of people who are multimorbid have both physical and mental health conditions, rising to >40% in the most deprived one-fifth of areas.^2^ People with comorbid physical and mental conditions have more complex care needs, and can find it more difficult to manage their conditions.^8^

Compared with people who are not multimorbid, people with multimorbidity require more input from the healthcare system. They require a higher number of GP consultations and have an increased likelihood of an emergency admission to hospital.^1^^,^^9^ There is, however, some evidence that, if a person is more able to manage their multiple health conditions independently, they have fewer emergency admissions.^9^^,^^10^ One study in an area of high deprivation showed that more time for complex consultations is associated with increased patient enablement, that is, ability to self-manage conditions.^11^ The Royal College of General Practitioners (RCGP), based on this premise, recommend longer consultations for patients with multimorbidity in order to reduce workload on the broader NHS.^12^ Likewise, people living with multimorbidity have identified longer primary care appointments as an optimal way of improving the quality of their care.^13^

Despite these recommendations, research in Scotland has shown that the greater need of patients with multimorbidity living in the most deprived quarter of areas is not reflected in longer consultation length. This contrasts with the least deprived quarter of areas where those with multimorbidity received longer consultations than those without.^14^ This is an example of the inverse care law, where the availability of good medical care tends to vary inversely with need, and can result in unmet need for health care. Research in Scotland has also demonstrated that, although consultation rates increase with deprivation, the social gradients in multimorbidity are much steeper, indicating potentially unmet need. The authors of the present study are not aware of any research examining whether this particular example of the inverse care law also applies in England, although consultation length has been found to be shorter in more deprived areas.^15^^,^^16^

**Table:** How this fits in

The vision of the Royal College of General Practitioners is that GPs will have more time to care for patients. Longer consultations are recommended where people need this, including people with multimorbidity. The risk of having multimorbidity is higher in more socioeconomically deprived areas. But, despite need being greatest in the most deprived areas of the UK, the number of GPs is falling fastest in these areas. Several studies have shown that GP consultations are shorter in more deprived areas. A study set in Scotland has shown that patients with multimorbidity had longer consultations with their GP, but only if they were living in the least deprived quarter of areas and not if they were living in the most deprived quarter of areas. The present study, set in England, confirms that consultations are shorter in more deprived areas. It also shows, however, that people with multimorbidity have longer consultations than those who do not have multimorbidity, and that this applies in both deprived and less deprived areas.

This research studied the association between GP consultation length and presence of multimorbidity or socioeconomic deprivation in England. It tested whether the difference in consultation length for patients with and without multimorbidity varied between more and less deprived areas in England. Whether these factors were affected by multimorbidity type was also assessed.

## METHOD

Data were obtained from the Clinical Practice Research Datalink (CPRD), a research database of anonymised patient records covering approximately 6.9% of the UK population.^17^ This dataset consisted of a random sample of people in England (*n* = 300 000) eligible for linkage to an area-based measure of socioeconomic deprivation, and who were registered between 1 April 2014 and 31 March 2016 (or died during this period) in an up-to-standard practice (that is, a quality indicator based on continuous recording of patient data and completeness of recorded deaths). Consultations over this 2-year follow-up period were included. For this study, those aged <18 years were excluded.

### Consultation duration

Consultation duration was captured in whole minutes and derived from the opening and closing time for a patient’s electronic patient record. Only face-to-face consultations with a GP or GP registrar were analysed. Consultations were excluded where the record was opened for administrative purposes, telephone consultations (owing to the large number which may be triage appointments followed by face-to-face consultations), or home visit consultations (as the recorded duration would only represent the time taken to record the consultation after it has ended). Consultations recorded as lasting >60 minutes were truncated at 60 minutes, as these were considered unlikely to reflect actual consultation length.^18^ Consultations recorded as lasting 0 minutes were set to 0.5 minutes.^15^

For the main analysis, consultations of duration <2 minutes were excluded, as it was deemed that these may not reflect accurate consultation length. In sensitivity analysis, all consultations were included irrespective of duration.

### Multimorbidity status

The presence or absence of 36 conditions was derived at the beginning of follow-up on 1 April 2014. These 36 conditions were identified in previous work because they are likely to be chronic, related to reduced quality of life and mortality risk, have substantial need for ongoing treatment,^1^ and use publicly available lists^19^ for Read codes (that is, codes used by UK primary care practitioners to record information about diagnoses) and product codes (that is, codes specific to CPRD to record information about pharmacological and non-pharmacological products). Patients were grouped according to the number of conditions they had. In addition, patients were grouped into: those with 0 or 1 condition; those with ≥2 conditions including ≥1 mental health condition (depression or anxiety, anorexia or bulimia, alcohol problems, other psychoactive substance use, schizophrenia), which are referred to as ‘multimorbid — including a mental health condition’; and those with ≥2 physical health conditions, which are referred to as ‘multimorbid — physical only’.

### Socioeconomic deprivation

Deprivation was based on the patient’s area of residence (Lower Layer Super Output Area level) using deciles of the 2015 Index of Multiple Deprivation (IMD)^20^ grouped into high deprivation (deciles 1–3), medium deprivation (4–7), or low deprivation (8–10). Linkage was undertaken by CPRD.

### Covariates

Patient and staff factors, which can influence consultation duration^15^ and may confound an association between duration and deprivation or multimorbidity, were included. Previous work shows females and older people tend to have longer consultations, though the association between duration and age is not linear.

More consultations may be used to extend the total consultation time where practice policies only allow for fixed, shorter appointments, so the number of consultations the patient had during the 2-year follow-up was adjusted for. GP registrars are GPs in training and are typically allocated longer duration for their consultations. GP registrars may also not be assigned the most complex patients. Consultations with female healthcare staff tend to be longer,^15^ as do consultations in urban areas.^20^

### Statistical analysis

Multilevel linear regression analysis was conducted with consultation length as the dependent variable. Patient sex and age, number of GP consultations in the 2-year follow-up period, GP trainee status, GP sex, urban–rural classification, IMD, and multimorbidity level were controlled for. Three-level regression models accounted for the non-independence of multiple consultations within patients, and patients within practices. Additionally, an interaction between IMD and multimorbidity was tested for.

Consultation length is not normally distributed, but previous studies^15^ have analysed it using means and multilevel linear regression models. In sensitivity analysis, the regression models were repeated using multilevel Poisson regression. The direction and statistical significance of the associations of interest were unchanged. Therefore, the linear regression results are presented here.

## RESULTS

The original sample of patients aged ≥18 contained data on 2 553 413 face-to-face consultations, of which 1 522 128 were with a GP or GP registrar. Of these, 263 209 lasted <2 minutes. The main analysis was conducted based on 1 258 919 consultations for 190 036 patients lasting ≥2 minutes. Consultations of duration <2 minutes were more common in those with more conditions (20.2% of those with ≥6 conditions and 13.3% of those with no conditions) (see Supplementary Table 1 for details).

**Table 1.** Characteristics of included patients (*N* = 190 036)

	**% (*n*)**
Sex	
Males	45.3 (86 106)
Females	54.7 (103 930)

Age (years)	
18–29	14.5 (27 551)
30–39	14.7 (27 913)
40–49	18.8 (35 699)
50–59	18.1 (34 483)
60–69	15.9 (30 399)
70–79	11.3 (21 383)
≥80	6.6 (12 608)

IMD	
Quintile 1 (least deprived)	25.9 (49 183)
Q2	21.1 (40 057)
Q3	20.3 (38 582)
Q4	18.4 (34 900)
Q5 (most deprived)	14.4 (27 314)

Multimorbidity level	
0 conditions	39.2 (74 548)
1 condition	25.3 (48 043)
2 conditions	14.9 (28 306)
3 conditions	9.0 (17 054)
4–5 conditions	8.4 (15 969)
≥6 conditions	3.2 (6116)

Multimorbidity type	
Not multimorbid^a^	64.5 (122 591)
Multimorbid — physical only^b^	23.1 (43 822)
Multimorbid — including a mental health condition^c^	12.4 (23 623)

a 0–1 long-term condition.

b ≥*2 physical conditions and no mental health conditions.*

c ≥*2 conditions with* ≥*1 mental health condition. IMD = Index of Multiple Deprivation. Q = quintile.*

Almost 55% of the sample were females, 25.9% lived in the least deprived fifth of areas in England, and 35.5% had ≥2 conditions (Table 1). A total of 23.1% had ≥2 physical conditions and 12.4% had multimorbidity that included ≥1 mental health condition.

In unadjusted analysis (Table 2), females had longer consultations (mean duration 11.0 minutes) and more consultations (mean 8.6 over 2 years) than males (mean 10.9 minutes and 6.7 consultations respectively). Older people did not have longer consultations, but they had more consultations compared with younger people. Compared with fully qualified GPs, GP registrars had longer consultations with a mean duration of 14.4 minutes. Mean consultation length was shorter for people living in the most compared with the least deprived fifth of areas (10.7 versus 11.2 minutes). Shorter consultations were also seen for patients who were not multimorbid (10.8 minutes compared with 11.0 for patients who are multimorbid). Among patients who are multimorbid, those with ≥1 mental health condition had mean consultation time of 11.1 minutes and those with only physical health conditions 10.9 minutes.

**Table 2.** Consultations by sociodemographic characteristics (*N* = 1 258 919)

		**Duration of consultations (includes consultations ≥2 minutes)**			**Number of consultations ≥2 minutes per patient over 2 years**	
	
		**Consultations (*N*)**	**Mean**	**SD**	**Mean**	**SD**
Sex	Males	492 383	10.9	7.8	6.7	7.9
	Females	766 536	11.0	7.8	8.6	9.1

Age (years)	18–29	139 667	10.6	7.4	5.7	6.2
	30–39	154 375	10.9	7.6	6.3	7.0
	40–49	204 351	11.2	7.7	6.5	7.1
	50–59	216 932	11.1	7.6	7.3	7.8
	60–69	220 044	10.9	7.7	8.6	8.9
	70–79	192 617	10.9	7.9	11.0	10.6
	≥80	130 933	10.7	8.9	13.0	13.7

GP registrar	No (Qualified GP)	1 150 219	10.6	7.5	—	—
	Yes	108 700	14.4	9.4	—	—

GP sex	Male	666 793	10.3	7.7	—	—
	Female	582 178	11.5	7.8	—	—
	Unknown	9948	12.1	8.9	—	—

Rural–urban classification	Rural	160 824	10.7	7.9	8.0	9.1
	Urban city	656 125	11.0	7.6	7.6	8.4
	Urban conurbation	441 970	11.0	8.0	7.8	8.8

IMD	Q1 (least deprived)	318 041	11.2	7.9	7.6	8.5
	Q2	258 653	11.0	7.8	7.5	8.8
	Q3	258 678	10.9	7.8	7.8	8.7
	Q4	236 385	10.8	7.8	7.9	8.5
	Q5 (most deprived)	187 162	10.7	7.6	8.0	78.5

Multimorbidity level	0 conditions	312 485	10.8	7.3	4.3	4.6
	1 condition	286 130	10.9	7.5	7.0	6.5
	2 conditions	218 395	10.9	7.7	9.2	8.1
	3 conditions	162 367	11.0	8.0	11.5	10.2
	4–5 conditions	187 229	11.0	8.2	14.4	13.1
	≥6 conditions	92 313	11.2	8.9	18.7	18.1

Multimorbidity type	Not multimorbid^a^	598 615	10.8	7.4	5.6	4.8
	Multimorbid	660 304	11.0	8.1	11.8	11.5
	Of which:					

	Multimorbid — physical only^b^	397 098	10.9	8.0	11.0	10.3
	Multimorbid — including a mental health condition^c^	263 206	11.1	8.3	13.4	13.2

a 0–1 long-term condition.

b ≥*2 physical conditions and no mental health conditions.*

c ≥*2 conditions with* ≥*1 mental health condition. IMD = Index of Multiple Deprivation. Q = quintile. SD = standard deviation.*

Table 3 summarises estimates from the regression models. In the main analysis limited to consultations lasting ≥2 minutes, controlling for patient and staff characteristics, residence in a more deprived area was associated with a shorter consultation. Mean duration was 0.46 (95% confidence intervals [CI] = 0.40 to 0.53) minutes shorter for those in the most compared with the least deprived fifth of areas. Consultation length increased with number of conditions the patient had and was 0.94 (CI = 0.84 to 1.03) minutes longer for those with ≥6 compared with no long-term conditions. Consultation length also depended on multimorbidity type, with patients with ≥2 physical conditions having 0.30 minutes longer with the GP and those with ≥2 conditions including a mental health condition having 0.47 minutes longer compared with patients who were not multimorbid (Table 4).

**Table 3.** Association^a^ between consultation duration and multimorbidity level and area deprivation

	**Consultations ≥2 mins, *N*= 1 258 919**		**All consultations, *N*= 1 522 128**	

	**Regression coefficient**	**CI**	**Regression coefficient**	**CI**
IMD				
Q1 (least deprived)	Ref		Ref	
Q2	−0.18	(−0.23 to −0.13)^b^	−0.12	(−0.17 to −0.06)^b^
Q3	−0.20	(−0.26 to −0.15)^b^	−0.12	(−0.18 to −0.07)^b^
Q4	−0.31	(−0.36 to −0.24)^b^	−0.23	(−0.29 to −0.17)^b^
Q5 (most deprived)	−0.46	(−0.53 to −0.40)^b^	−0.27	(−0.34 to −0.21)^b^

Multimorbidity level				
0	Ref		Ref	
1	0.07	(0.02 to 0.12)^c^	−0.08	(−0.13 to −0.03)^c^
2	0.22	(0.17 to 0.28)^b^	0.01	(−0.05 to 0.07)
3	0.45	(0.38 to 0.52)^b^	0.21	(0.14 to 0.28)^b^
4–5	0.67	(0.60 to 0.74)^b^	0.45	(0.37 to 0.52)^b^
≥6 conditions	0.94	(0.84 to 1.03)^b^	0.77	(0.66 to 0.87)^b^

a Three-level regression model (consultations nested within patients within practices) includes patient sex, age, number of consultations per year, GP trainee status, GP sex, urban–rural classification, multimorbidity level, and IMD.

b P<*0.001.*

c P<*0.05. CI = confidence interval. IMD = Index of Multiple Deprivation. Q = quintile. Ref = the category against which the other categories are compared in the statistical model.*

**Table 4.** Association^a^ between consultation duration and multimorbidity type and area deprivation

	**Consultations ≥2 mins *N*= 1 258 919**		**All consultations *N*= 1 522 128**	

	**Regression coefficient**	**CI**	**Regression coefficient**	**CI**
IMD				
Q1 (least deprived)	Ref		Ref	
Q2	−0.18	(−0.23 to −0.12)^e^	−0.11	(−0.17 to −0.06)
Q3	−0.19	(−0.25 to −0.14)^e^	−0.11	(−0.17 to −0.06)^f^
Q4	−0.29	(−0.35 to −0.23)^e^	−0.22	(−0.27 to −0.16)^e^
Q5 (most deprived)	−0.45	(−0.51 to −0.38)^e^	−0.26	(−0.32 to −0.19)

Multimorbidity type				
Not multimorbid^b^	Ref		Ref	
Multimorbid — physical only^c^	0.30	(0.25 to 0.35)^e^	0.19	(0.14 to 0.24)^e^
Multimorbid — including a mental health condition^d^	0.47	(0.42 to 0.53)^e^	0.29	(0.24 to 0.35)^e^

a Three-level regression model (consultations nested within patients within practices) includes patient sex, age, number of consultations per year, GP trainee status, GP sex, urban–rural classification, multimorbidity type, and IMD.

b 0–1 long-term condition.

c ≥*2 physical conditions and no mental health conditions.*

d ≥*2 conditions with* ≥*1 mental health condition.*

e P<*0.001.*

f P<*0.05. CI = confidence interval. IMD = Index of Multiple Deprivation. Q = quintile. Ref = the category against which the other categories are compared in the statistical model.*

**Figure 1.**
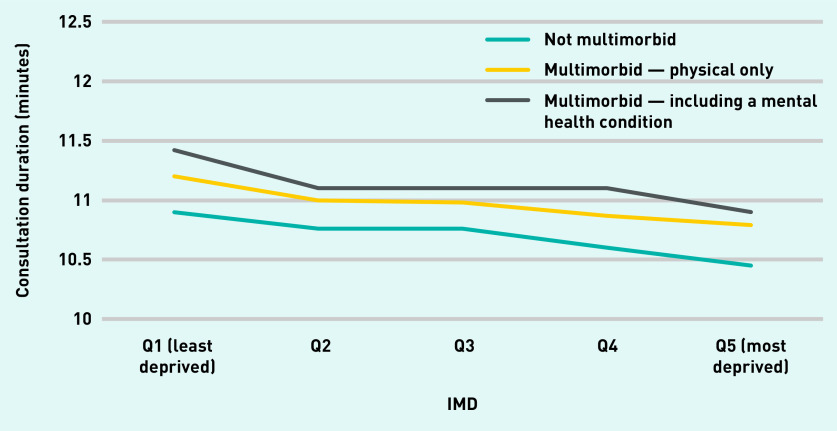
***Consultation duration by Index of Multiple Deprivation and multimorbidity type: consultations lasting ≥2 minutes.^a^*** ***^a^Not multimorbid: 0–1 long-term condition; multimorbid — physical only: ≥2 physical conditions and no mental health conditions; multimorbid — including a mental health condition: ≥2 conditions with ≥1 mental health condition. IMD = Index of Multiple Deprivation. Q = quintile.***

No clear evidence was found that the association between multimorbidity level or type and consultation length was different for patients in more versus less deprived areas. Figure 1 shows consultation duration by IMD and multimorbidity type from the model, including the interaction of these two factors. It illustrates that, for all multimorbidity types, patients in the most deprived areas had shorter consultations than those in the least deprived areas. It also illustrates that the mean consultation length for a patient without multimorbidity in a low deprivation area (10.9 minutes) was the same as that for a multimorbid patient with physical and mental health conditions in an area of high deprivation.

The same patterns were found when all consultations (including those lasting >2 minutes) were analysed. Regression estimates show smaller differences in consultation length by multimorbidity level when all consultations were included. This was expected because very short consultations were more common in patients with more long-term conditions.

## DISCUSSION

### Summary

Living in an area of high socioeconomic deprivation is associated with shorter GP consultations. GP consultation length increased with increasing number of health conditions. Consultations were also longer for multimorbid patients with a mental health condition than for multimorbid patients with physical conditions only. The positive association between consultation length and number of health conditions was seen in both deprived and less deprived areas.

### Strengths and limitations

A strength of this study was the large sample size and the use of routine data to minimise selection bias. The use of multilevel regression analysis allowed for unobserved similarities between practices and between patients that could affect consultation duration. The association between deprivation and duration remained on adjustment for total number of consultations, indicating that use of additional consultations did not explain the shorter consultations in more deprived areas. This study was limited by several factors. CPRD data provide consultation time based on the open and close time of the electronic record. This is the amount of time a practitioner had the file open, which may be affected by other factors including practitioner preference regarding whether to complete and close a record later in the day or while the patient is present, and the possibility of clinicians forgetting to close a consultation until after the care episode has ended (although all consultations were capped at a maximum of 60 minutes). There is, however, no evidence to suggest that these factors differ by patient level of deprivation or multimorbidity. Previous analysis used video-recording to accurately capture consultation duration, although this approach may have altered GP behaviour. This study also focused on primary care delivered by GPs. Future analysis should also consider consultations with nurses, because nurses provide a sizeable proportion of primary care for people with multimorbidity.^18^ As primary care only forms a single component of health care for people with multimorbidity, further studies should also consider care provided within hospitals and other parts of the health system.

The adjusted difference in consultation time for patients in the most compared with the least deprived areas amounted to 0.5 minutes. The magnitude of this difference appears small, and further work is needed to quantify associations between consultation length and patient experience, outcomes, or use of other health services, as others have noted.^21^ This small value should, however, be interpreted in the context of an average consultation of just under 11 minutes.

### Comparison with existing literature

This paper adds to the evidence that multimorbidity and deprivation influence consultation time with a GP. Particularly concerning is ongoing evidence indicating that patients in deprived areas have shorter consultation times.^15^ These findings support evidence previously found in Scotland based on video-recorded consultations to provide an accurate measure of time spent with patients.^14^ That study considered a single consultation for each patient, whereas the present study adds to their findings in showing that a similar pattern is observed (that is, shorter consultations for patients in more deprived areas) across multiple consultations over a 2-year period of usual care. This is likely to reflect ongoing job pressures for GPs in deprived areas, and a greater need for care among this group of patients. Practices in deprived areas tend to have lower levels of GP staffing.^22^ The staffing level and patient load at a particular GP surgery influences the work pressure for GPs, and can therefore influence the consultation time available.^22^ Given these pressures, other factors that could affect patient experience and patient outcomes, such as continuity of care or GP empathy, may also differ by deprivation level, as has been found in Scotland.^15^ These other characteristics of the consultation were not examined, and further work is needed to explore those factors and to test the contribution to outcomes of consultation length, continuity of care, and patient experiences of the consultation. The previous study set in Scotland compared consultation length for practices in high- and low-deprivation areas, whereas the present study used deprivation in the patient’s local area. Although patient and practice deprivation will be positively correlated, they may influence consultation length independently via different mechanisms. Further analysis including deprivation at both patient and practice level would be useful, but was not possible with the current data.

It was also identified that patients with multimorbidity receive longer consultations. Consultation length increased with the number of conditions a patient had. This is in line with calls for longer GP consultations for patients who are multimorbid; however, whether these relatively small differences in consultation length are related to, or sufficient to achieve, better patient outcomes remains to be tested. Previous evidence from Scotland^14^ showed that patients with multimorbidity received around 3 minutes longer with their GP than those without multimorbidity in affluent areas, but that this was not the case in deprived areas. In England, patients with multimorbidity in both more and less deprived areas had longer consultations than their non-multimorbid counterparts.

GPs in England are spending longer with patients who have more long-term conditions. The consultation length also depends on the types of conditions the patient has. Having a mental health condition can make it more difficult to manage complex care needs, and longer consultations have been linked to better handling of psychological problems in primary care.^23^^,^^24^ This analysis shows that multimorbidity including a mental health condition was associated with having a longer consultation compared with having multiple physical conditions and compared with not having multimorbidity. However, the analysis also shows that this additional time is counteracted by living in a deprived area. The association between deprivation and consultation length is equal in magnitude and opposite in effect to the association between multimorbidity and consultation length. This means that a patient with multiple mental and physical health conditions living in an area of high deprivation receives the same amount of time with their GP as a person without multimorbidity in an area of low deprivation.

### Implications for research and practice

This study shows that the inverse care law is alive and well in general practice in England. Not only do people living in more deprived areas of the country have on average shorter GP appointments, the same pattern is observed even when those people have multiple health conditions. This suggests that there could be unmet need among patients with complex care needs, particularly patients living in deprived areas with both mental and physical health conditions.

Understanding *why* shorter consultation times in general practice are observed in areas of high deprivation is crucial to understanding *how* this could be changed. This includes understanding patient factors as well as those related to the organisation and delivery of general practice.

Undersupply of GPs relative to population need and corresponding higher workload may be a key driver of shorter consultation times, and evaluation of the impact of initiatives encouraging GPs to train and work in underdoctored areas is awaited. Increasing skill mix in primary care by recruiting additional allied health professionals is seen as one way of freeing up GP time to focus on more complex patients. These staff may also directly contribute to and improve care for people with multimorbidity. Initiatives to ensure these additional staff will be distributed equitably across the country and to encourage them to work in areas of high deprivation will be needed. If additional staff gravitate to areas of lower deprivation, then there will be paradoxically even fewer staff relative to need in the areas of highest deprivation.^22^

The positive association between consultation length and number of long-term conditions that were identified is in line with calls for longer GP consultations for patients who are multimorbid, although whether these relatively small differences in consultation length are related to, or sufficient to achieve, better patient outcomes remains to be tested.
